# Investigating the role of cytochrome *bd* oxidases in the antibacterial activity of madecassic acid and derivatives thereof

**DOI:** 10.1039/d5md01116g

**Published:** 2026-01-13

**Authors:** Samantha A. Henry, Geraud N. Sansom, Thao Thi Phuong Tran, Ryan A. Boughton, Guy Joiner, Calum M. Webster, H. Ireshika C. de Silva, Michelle D. Garrett, Christopher J. Serpell, Gary K. Robinson, Mark Shepherd

**Affiliations:** a School of Natural Sciences, University of Kent Canterbury CT2 7NH UK M.Shepherd@kent.ac.uk; b Institute of Chemistry, Vietnam Academy of Science and Technology 18 Hoang Quoc Viet Road Nghia Do Hanoi Vietnam; c Department of Chemistry, University of Colombo Colombo 03 Sri Lanka; d School of Pharmacy, University College London 29-39 Brunswick Square London WC1N 1AX UK chris.serpell@ucl.ac.uk

## Abstract

Natural products are valuable starting points for drug discovery, although individual modes of action are often difficult to pin down. Ursanes such as madecassic acid have been shown to have antibacterial properties, but a variety of mechanisms have been proposed. In this paper, we report previously uninvestigated activity against cytochrome *bd* oxidases which are only found in prokaryotes and are therefore promising new targets, using madecassic acid and a set of synthetically modified derivatives. Our work shows that madecassic acid and its derivatives can block activity of these enzymes, while phenotypic effects in membrane and whole organism assays are more complex, consistent with modulation of multiple pathways depending upon molecular structure. This provides a new route to ursane-based antibacterial action while highlighting the importance of chemical modifications in fine-tuning biological activity of natural products.

## Abstract 2

Madecassic acid is found to have activity against cytochrome proteins found only in bacteria, offering a new route to antibiotics.

## Graphical abstract

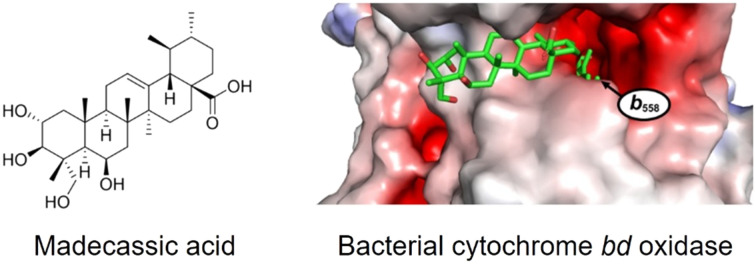

## Introduction

Ursanes are pentacyclic terpenoids which have been shown to have antibacterial properties.^1–4^ Madecassic acid, an ursane produced by the common Asian herb *Centella asiatica*, has been shown to have good activity against *S. aureus* and moderate activity against other bacterial species, with evidence consistent with a mechanism including increasing membrane and wall permeability, interaction with DNA, and inhibition of protein synthesis.^5^ This is similar to evidence for ursolic acid, a related compound also found to be active against Gram-positive bacteria.^6^ Unpurified extracts of *C. asiatica* have shown to be active against Gram-positive and -negative bacteria.^7^ These trends have been demonstrated across a range of modified ursanes,^8^ and the potential of asiatic acid to inhibit bacterial growth had led to its incorporation into gel materials to aid *in vivo* wound healing.^9^

Cytochrome *bd* oxidases are attractive drug targets for discovery of new antibacterials, since they are found only in prokaryotic organisms,^10^ and therefore permit specific killing of bacteria. The proteins are found on the inner membrane, where their role is to couple reduction of molecular oxygen with oxidation of quinols, resulting in a proton motive force used in the generation of ATP. Blocking cytochrome *bd* oxidase activity would therefore impede the capacity of bacteria to access energy required for growth.^11^*Escherichia coli*, a Gram-negative bacterium responsible for many urinary tract and bloodstream infections, has two *bd* oxidases: *bd*-I which is expressed in microaerobic environments, and *bd*-II which is predominant in anaerobic conditions.^12^

We have discovered effective steroidal ligands for *bd*-I which inhibit growth and provide lethality against bacteria, with the strongest measured affinities in *E. coli*.^13^ Since ursane-type structures resemble sterols, it is possible that binding of bacterial cytochrome oxidases is a key component in the antibacterial mechanism of action of the compound class. Since literature reports that a number of mechanisms are at play, it would also be useful to know if the balance of these processes can be modulated through structural refinement of the compounds. In this paper we test madecassic acid (**MA**) and three synthetic analogues for cytochrome oxidase inhibition through molecular docking, measurement of oxygen consumption in isolated membranes, and testing against live *E. coli* models, each engineered to have access to different oxidases.

## Experimental procedures

### Chemical synthesis

Madecassic acid (**MA**) was obtained by extraction of leaves grown in Quang Tho II Commune, Hue Province, VietNam, following our reported precedure.^14^ Deuterated solvents were purchased from Cambridge Isotope Laboratories Inc. Solvents and reagents were purchased from Thermo Fisher Scientific, Fluorochem, or Alfa Aesar.

A Bruker AVII 400 MHz spectrometer was used to record NMR spectra, and each spectrum was calibrated to the known chemical shift of the residual solvent peak of the deuterated solvent used. Chemical shifts were reported in part per million (ppm) and J coupling values were reported in Hz. Proton NMR spectra were obtained at 400 MHz and ^13^C spectra were obtained at 101 MHz. Spectral data was processed using MestReNova software. Electrospray mass spectrometry data was obtained using a Thermo MSQPlus instrument fitted with a Zorbax SB-C18 5 μm 3.0 × 150 mm column using H_2_O + 0.1% formic acid and MeOH + 0.1% formic acid or H_2_O + 0.1% TFA and MeCN + 0.1% TFA mobile phases. Data was analysed using Chromeleon™ Chromatography Data System (CDS) Software. A Bruker micrOTOF-Q LCMS system was used to obtain high-resolution mass spectrometry data, samples were dissolved in HPLC-grade methanol and injected using direct injection mode with a mobile phase system of 50 : 50 MeOH and H_2_O. Data was processed using Bruker Compass Hystar software.

#### MA-2

**MA** (201.6 mg, 0.40 mmol) was dissolved in 6 mL of dry pyridine under nitrogen and acetic anhydride (0.13 mL, 1.40 mmol) was injected into the reaction slowly. After 24 hours, 10 mL pyridine was added, and additional acetic anhydride (4 mL, 42.3 mmol) was added to the reaction. After 48 hours, TLC (after staining with vanillin) showed complete consumption of starting material. The reaction was transferred to a separating funnel, EtOAc (25 mL) was added, and the reaction organic layer was washed with 1 M HCl_(aq)_ (3 × 50 mL). The organic layer was dried over anhydrous MgSO_4_, filtered and the solvent removed under reduced pressure. Crude product was purified using gradient silica gel column chromatography (0–50%, EtOAc : CH_2_Cl_2_) giving **MA-2** as a white needle crystalline solid (183 mg 72.6% yield). ^**1**^**H NMR** (400 MHz, CDCl_3_) *δ* 5.27 (d, *J* = 3.6 Hz, 1H), 5.22 (td, *J* = 11.3, 4.8 Hz, 2H), 5.00 (d, *J* = 10.3 Hz, 1H), 4.33 (d, *J* = 3.8 Hz, 1H), 3.93 (d, *J* = 11.9 Hz, 1H), 3.70 (d, *J* = 12.0 Hz, 1H), 2.19 (d, *J* = 11.2 Hz, 1H), 2.15–0.80 (m, 46H). ^**13**^**C NMR** (101 MHz, CDCl_3_) *δ* 183.9, 171.0, 170.6, 170.6, 137.3, 125.7, 75.0, 70.0, 68.0, 65.5, 52.5, 48.3, 48.0, 47.9, 45.9, 42.5, 40.8, 39.2, 38.9, 38.7, 37.4, 36.7, 30.7, 28.0, 24.2, 23.6, 23.4, 21.3, 21.2, 21.2, 21.0, 20.9, 18.8, 18.6, 17.1, 15.5, 14.3. **HREI-MS**: *m*/*z* calculated for C_36_H_54_O_9_Na [M + Na^+^] 653.3660; observed 653.3672.

#### MA-3

The *tert*-butyloxycarbonyl-protected version of **MA-3** (**MA-3Boc**) was first synthesised. HATU (131.1 mg, 0.334 mmol) and **MA-2** (175.5 mg, 0.278 mmol) were dissolved in 5 mL dry CH_2_Cl_2_ and DIPEA (0.145 mL, 0.835 mmol) was slowly added to the reaction and it was stirred for 1 hour. *Tert*-butyl 10-aminodecylcarbamate (152.8 mg, 0.556 mmol) was dissolved in dry CH_2_Cl_2_ (5 mL) and slowly added to the reaction and it was left to stir overnight. After 22 hours, the reaction was transferred to a separating funnel and diluted with CH_2_Cl_2_ (30 mL). It was then extracted with 1 M HCl_(aq)_ (3 × 50 mL) and brine (1 × 50 mL). Dried over anhydrous MgSO_4_, filtered and reduced under vacuum. The crude product was purified by isocratic column chromatography (25% : 75%, EtOAc : CHCl_3_) to yield **MA-3Boc** as a white solid (236 mg, 95.8%). ^**1**^**H NMR** (400 MHz, CDCl_3_) *δ* 5.85 (t, *J* = 5.4 Hz, 1H), 5.34 (t, *J* = 3.5 Hz, 1H), 5.22 (td, *J* = 11.0, 4.7 Hz, 1H), 5.01 (d, *J* = 10.3 Hz, 1H), 4.57 (s, 1H), 4.34 (t, *J* = 3.8 Hz, 1H), 3.94 (d, *J* = 11.9 Hz, 1H), 3.71 (d, *J* = 12.0 Hz, 1H), 3.23 (dq, *J* = 13.3, 6.8 Hz, 1H), 3.14–2.93 (m, 3H), 2.17–0.77 (m, 72H). ^**13**^**C NMR** (101 MHz, CDCl_3_) *δ* 177.9, 170.9, 170.5, 170.5, 139.4, 125.5, 79.1, 75.0, 70.1, 67.4, 65.4, 54.2, 48.1, 47.9, 47.8, 46.0, 43.1, 42.5, 40.6, 39.9, 39.6, 39.2, 38.8, 38.8, 37.4, 37.3, 31.0, 30.2, 29.5, 29.5, 29.3, 29.3, 29.3, 29.2, 28.5, 27.9, 27.2, 26.9, 24.9, 23.5, 23.3, 21.3, 21.2, 21.0, 20.9, 18.7, 18.3, 17.3, 15.4. **HREI-MS**: *m*/*z* calculated for C_51_H_84_N_2_O_10_Na [M + Na^+^] 907.6018; observed 907.6052.

**MA-3Boc** (131.2 mg, 0.148 mmol) was then dissolved in CH_2_Cl_2_ (3.7 mL), 4 M HCl in dioxane (3.7 mL, 14.8 mmol) was added to the solution at 0 °C. The reaction was stirred and left to warm to room temperature overnight. After 21 hours, solvent was removed under reduced pressure. The residue was washed into a separating funnel with a mixture of CH_2_Cl_2_ (50 mL) and sat. NaHCO_3(aq)_ (50 mL) and aqueous layer removed. It was then extracted with a further sat. NaHCO_3(aq)_ (2 × 50 mL) and brine (1 × 50 mL). Dried over anhydrous MgSO_4_, filtered and reduced under vacuum. Extracted product was clean by NMR and required no further purification yielding **MA-3** (105.8 mg, 90.9%). ^**1**^**H NMR** (400 MHz, CDCl_3_) *δ* 5.92 (t, *J* = 5.4 Hz, 1H), 5.34 (d, *J* = 3.7 Hz, 1H), 5.21 (td, *J* = 10.9, 4.5 Hz, 1H), 5.00 (d, *J* = 10.3 Hz, 1H), 4.83 (s, 2H), 4.31 (d, *J* = 5.1 Hz, 1H), 3.94 (d, *J* = 11.7 Hz, 1H), 3.70 (dd, *J* = 12.5, 2.6 Hz, 1H), 3.28–2.94 (m, 3H), 2.85 (s, 2H), 2.25–0.73 (m, 62H). ^**13**^**C NMR** (101 MHz, CDCl_3_) *δ* 178.1, 171.1, 170.7, 170.6, 139.4, 125.5, 75.0, 70.2, 67.2, 65.4, 54.1, 48.1, 47.9, 47.8, 46.0, 43.8, 43.1, 42.5, 40.4, 39.9, 39.7, 39.2, 38.8, 37.4, 37.3, 31.0, 29.5, 29.4, 29.3, 29.3, 29.2, 29.1, 27.8, 27.1, 26.7, 24.9, 23.5, 23.3, 21.3, 21.3, 21.0, 20.9, 18.7, 18.3, 17.3, 15.4.

#### MA-4

**MA-3** (52.0 mg, 0.0662 mmol) was dissolved in MeOH (5 mL). 4 M NaOH_(aq)_ solution (0.080 mL, 0.331 mmol) was injected into the reaction, and it was left to stir overnight. After 20 hours, reaction was transferred to a separating funnel, EtOAc (50 mL) was added. The organic layer was extracted with 1 M NaOH_(aq)_ (3 × 50 mL). Dried over anhydrous MgSO_4_, filtered and reduced under vacuum. Extracted product was pure and required no further purification, yielding **MA-4** (43.6 mg, 99.9%). ^**1**^**H NMR** (400 MHz, methanol-*d*_4_) *δ* 5.39 (t, *J* = 3.6 Hz, 1H), 4.37 (t, *J* = 3.4 Hz, 1H), 3.74 (ddd, *J* = 11.3, 9.3, 4.3 Hz, 1H), 3.58 (d, *J* = 11.1 Hz, 1H), 3.43 (d, *J* = 11.1 Hz, 1H), 3.30 (d, *J* = 8.0 Hz, 1H), 3.21–3.01 (m, 2H), 2.75–2.66 (m, 2H), 2.20–0.79 (m, 54H). ^**13**^**C NMR** (101 MHz, methanol-*d*_4_) *δ* 180.1, 139.7, 127.1, 78.0, 69.6, 68.3, 65.7, 54.3, 50.2, 49.1, 48.9, 44.8, 43.9, 42.0, 41.2, 40.9, 40.8, 40.3, 40.1, 38.8, 38.5, 32.2, 32.0, 30.7, 30.7, 30.7, 30.6, 30.5, 30.3, 28.9, 28.3, 27.9, 25.2, 24.5, 24.1, 21.6, 19.3, 19.3, 17.7, 15.3. **HREI-MS**: *m*/*z* calculated for C_40_H_71_N_2_O_5_ [M + H^+^] 659.5357; observed 659.5392.

### Computation and molecular docking

Ligand docking to an AlphaFold2 model of *E. coli* cytochrome *bd*-I was performed essentially as described in detail previously.^13^ Briefly, preparation of *in silico* ligand and protein files was performed using AutoDockTools and PyMOL.^15^ The equation *K*_d_ = exp(Δ*G*/(*R* × *T*)) with Δ*G* = binding energy (kcal mol^−1^), *R* = gas constant = (1.986 cal mol^−1^ K^−1^) and *T* = temperature (298 K) was used to estimate the dissociation constants.

Clog *P* values were calculated using the Chemical Properties tool in Signals ChemDraw v23.1.2.7.

### Bacterial strains

EC958 is a multidrug-resistant *E. coli* O25: H4-ST131 isolate.^16,17^ Generation of the EC958 *bd*-I only strain (genotype Δ*cyoA appCB*::Cm) is described elsewhere.^13^

### Growth assays

Starter cultures were grown in 10 mL LB (*E. coli*) in sterile 50 mL conical flasks at 180 rpm and 37 °C until stationary phase was reached and were used to inoculate 50 mL of fresh growth medium in 250 mL conical flasks. M9 minimal medium was used for *E. coli* (16 g L^−1^ Na_2_HPO_4_·2H_2_O, 3 g L^−1^ KH_2_PO_4_, 0.5 g L^−1^ NaCl, 1 g L^−1^ NH_4_Cl, 0.24 g L^−1^ MgSO_4_, 0.01 g L^−1^ CaCl_2_, 0.1% casamino acids and 2% glycerol). Drug stocks were prepared in DMSO so that their final concentrations were 40× higher than the working concentrations. Greiner F-bottom sterile 96-well plates were prepared by adding 100 μL of a 2× concentrated growth medium, 61.7 μL sterile milliQ H_2_O, 5 μL of the drug and 33.3 μL of cell culture (final OD_600_ of ∼0.1). Cells were grown in a FLUOstar Omega plate reader at double orbital pattern setting.

### Oxygen consumption assays

Membrane preparation and oxygen consumption were performed as described previously.^13^ Briefly, cells were grown to exponential phase then harvested *via* centrifugation for 20 min at 4000 rpm and 4 °C. The cell pellet was resuspended in ice-cold sonication buffer (20 mM Tris/HCl at pH 7.4, 2 mM MgCl_2_, and 1 mM EGTA). The resuspended cells were sonicated (6 × 30s on ice at 15 μM) before centrifugation at 44 000 rpm for 1 h and 4 °C to isolate membranes. The membrane pellet was resuspended in 20 mM Tris/HCl (pH 7.4) at a final concentration of 100 mg mL^−1^ and stored at −20 °C. For oxygen measurements a Rank Brothers oxygen electrode with a 4 mL closed chamber was used at 37 °C, which contained 50 mM HEPES pH 7.4, 0.5 mg mL^−1^ membranes (based on wet membranes) and DMSO-solubilised drug (added from 40× final concentration). A final concentration of 8 mM succinate (pH 7.4) was added (from 160 mM stock) to initiate the reaction with a single run lasting 15–20 min.

### Viability assays

Viability assays were performed essentially as described previously.^13^ Briefly, *E. coli* was grown overnight in 10 mL LB medium and was used to inoculate M9 medium. 20 μL of each drug was added to the wells of row A of a 96 well plate followed by 180 μL of cells (OD_600_ of 0.1). Cells were exposed to drug for 3 hours at 37 °C. Following drug exposure, serial dilutions were performed in 1 × phosphate buffered saline (pH 7.4) before being spotted onto LB agar plates overnight to determine changes in cell survival. Six repeats were performed for each concentration of drug which included two biological repeats and three technical repeats of each.

## Results

**Fig. 1:**
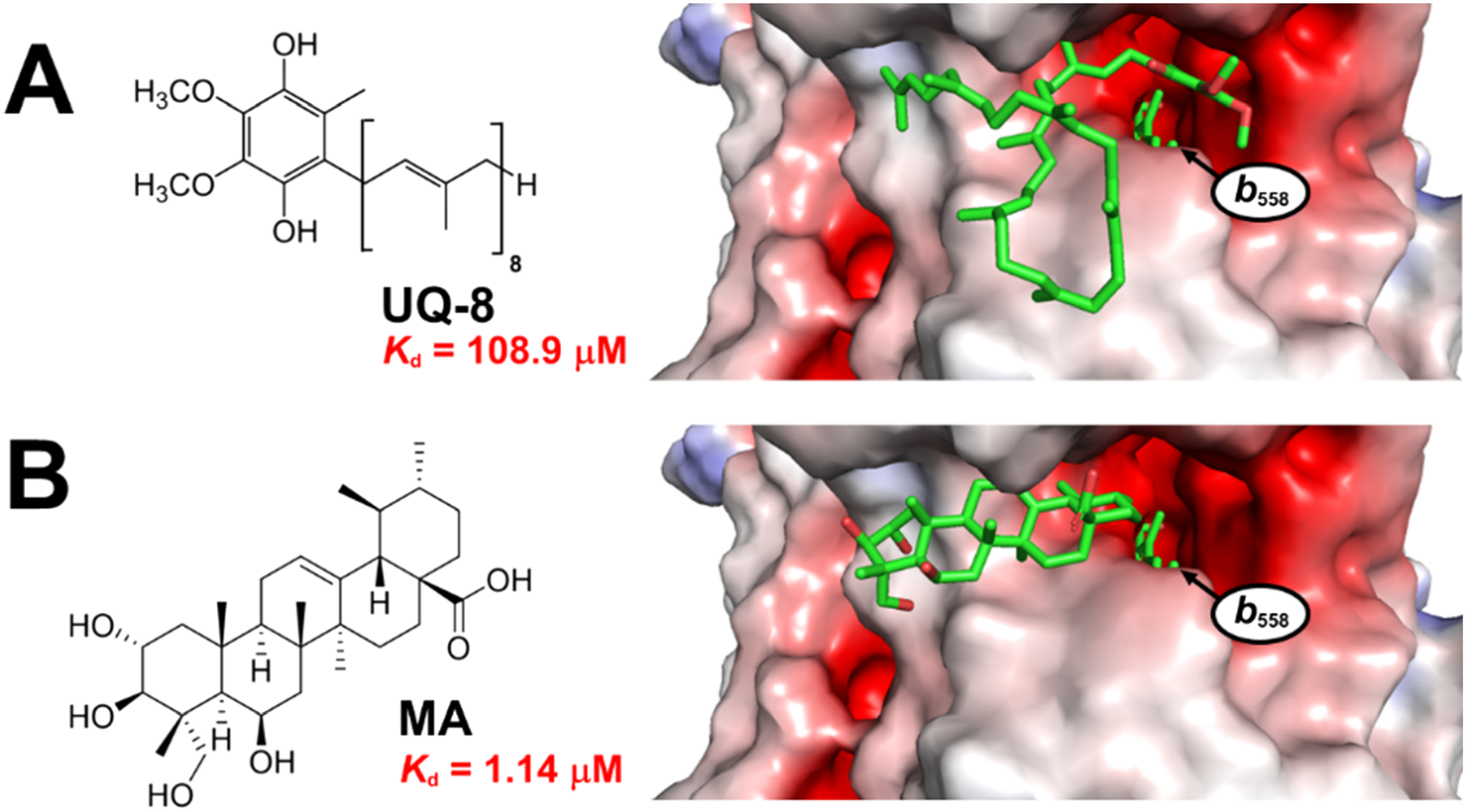
Docking of ubiquinol-8 (A) and **MA** (B) to the quinol-binding site of an AlphaFold 2 model for *E. coli* cytochrome *bd*-I. Haem *b*_558_ is a cytochrome *bd*-I cofactor that accepts electrons from the ubiquinol substrate, and is labelled on the figure. Predicted affinities from Autodock Vina are shown in red.

Both **MA** and ubiquinol-8 (**UQ-8**) the natural substrate of cytochrome *bd*-I were docked to the quinol site of the *E. coli* AlphaFold2 cytochrome *bd*-I structural model.^13^ The binding affinity values predicted by AutoDock Vina (Fig. 1) indicate that **MA** is substantially more tightly bound by cytochrome *bd*-I (1.14 μM) than **UQ-8** (108.9 μM). The carboxylic acid of **MA** was found to reside in the hydrophobic cleft (red, Fig. 1) close to where the aromatic unit of **UQ-8** sits, while the pentacyclic hydrocarbon occupies a similar space to that of the oligoisoprene tail of **UQ-8**. The polar units on **MA** point out of the protein cavity towards the aqueous environment.

**Fig. 2:**
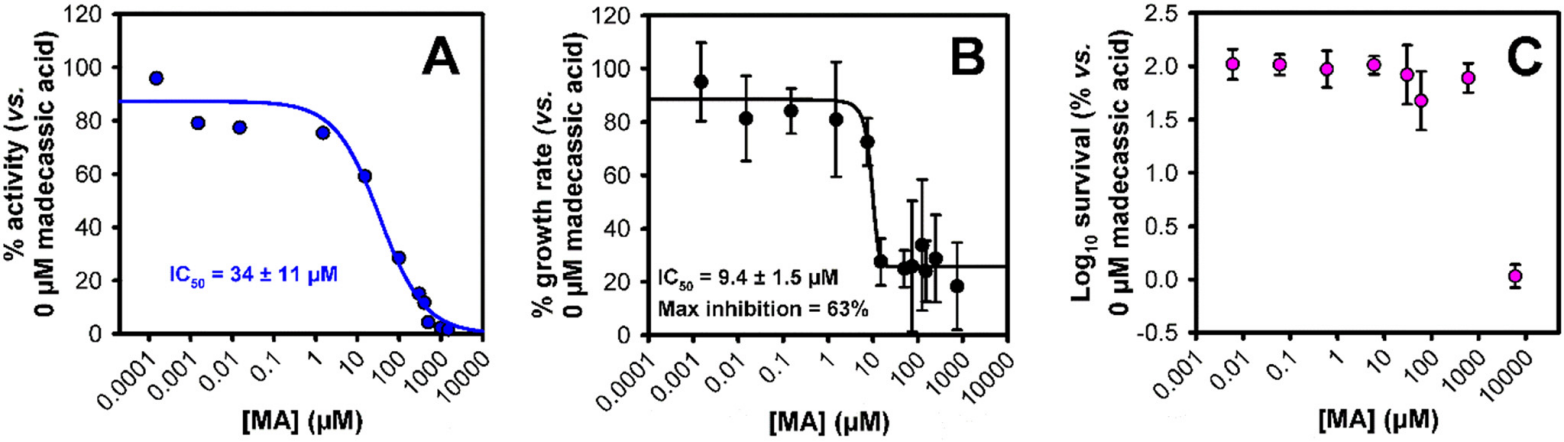
Impact of madecassic acid upon cytochrome *bd*-I activity, *E. coli* growth, and *E. coli* survival. (A) Dose inhibition curve of madecassic acid against oxygen consumption activity of *E. coli* EC958 cytochrome *bd*-I-only membranes. (B) Growth assay of **MA** against *E. coli* cytochrome *bd*-I only cells. (C) Survival assay of **MA** against *E. coli* EC958 cytochrome *bd*-I only cells. Dose response data were fitted to three- or four-parameter logistic equations using nonlinear regression (Sigmaplot) to generate IC_50_ values with standard error. Error bars represent standard deviations for at least four technical repeats, including two biological repeats.

These predictions suggest that **MA** would be able to competitively inhibit cytochrome *bd*-I. To test this prediction, we performed oxygen consumption assays to measure oxidoreductase activity using isolated *E. coli* EC958 cytochrome *bd*-I only membranes (Fig. 2A),^13^ and **MA** obtained by extraction of *C. asiatica*,^14^ resulting in an IC_50_ of 34 ± 11 μM. This activity is consistent with the docking results in that **MA** would be expected to outcompete **UQ-8**. On this basis, we advanced to assessing the ability of **MA** to inhibit the growth of cytochrome *bd*-I only *E. coli* cells (Fig. 2B), giving an IC_50_ of 9.4 ± 1.5 μM, with a maximum inhibition of 63%. We further conducted a viability assay (Fig. 2C) which showed that **MA** did not kill *bd*-I only *E. coli* cells. The observed lack of lethality may relate to permeability of the cell membrane and/or wall to **MA**, but is consistent with other tests on Gram-negative bacteria.^5^

To investigate the contribution of cytochrome *bd*-I to **MA**-mediated respiratory inhibition, growth experiments were also performed on the wild type EC958 strain, which encodes an additional respiratory oxidase cytochrome *bo*′ that is expressed under the aerobic conditions tested (Fig. S1A). These data revealed an IC_50_ of ∼10 μM and maximal inhibition of 48%, which is consistent with the wild type responding to respiratory inhibition in a similar way to the *bd*-I only strain. In addition, the wild type strain was not susceptible to **MA**-mediated killing at concentrations below 1 mM (Fig. S1B), confirming that **MA** is bacteriostatic but not bactericidal towards both strains.

**Fig. 3:**
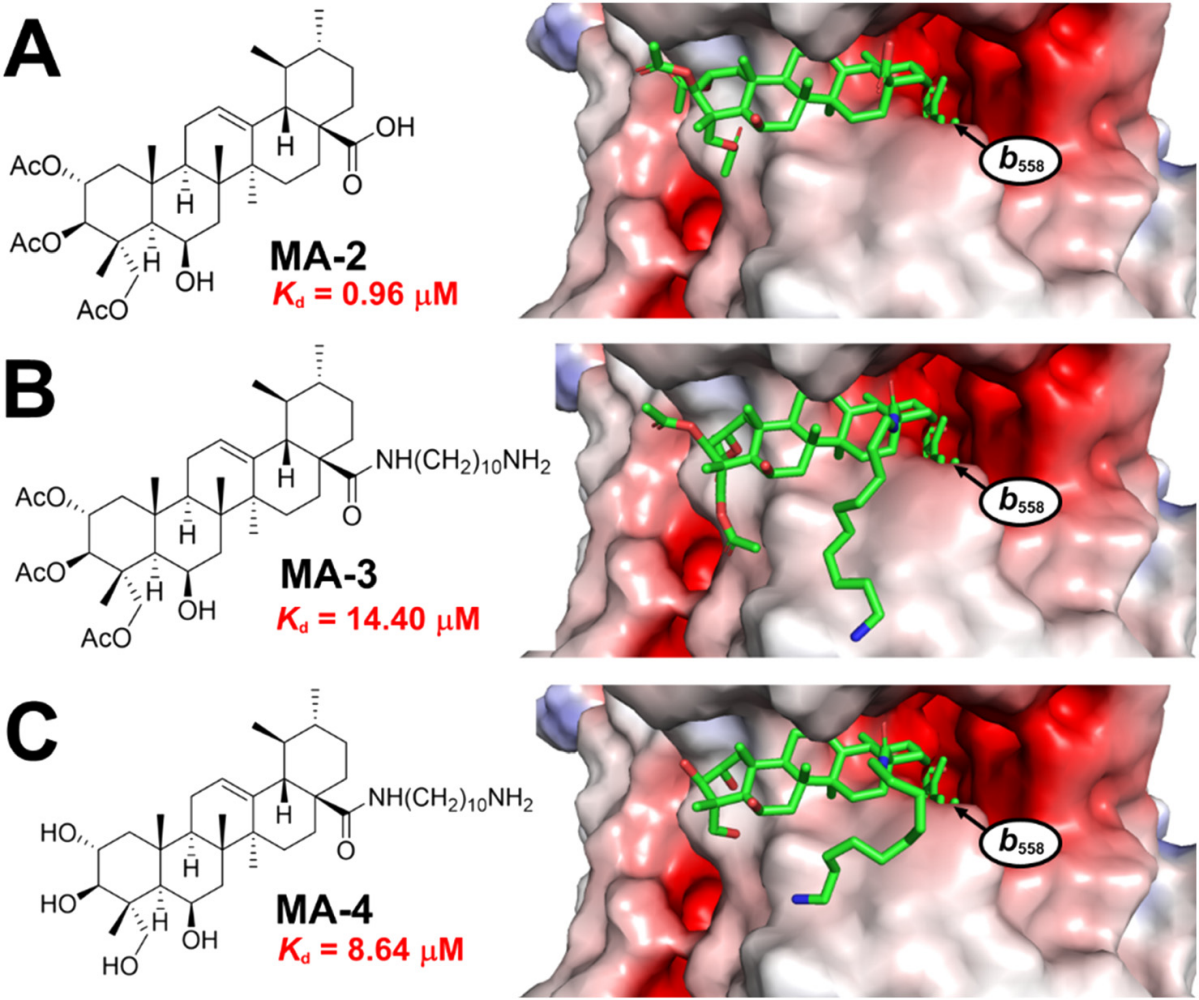
Madecassic acid derivatives docked to the quinol binding site of an AlphaFold 2 model for *E. coli* cytochrome *bd*-I. Haem *b*_558_ is a cytochrome *bd*-I cofactor that accepts electrons from the natural substrate ubiquinol (labelled as *b*_558_). (A) Docking of **MA-2**. (B) Docking of **MA-3**. (C) Docking of **MA-4**.

We then decided to explore variations upon the MA skeleton which might alter access to proteins on the bacterial inner membrane. For this reason, we examined modified versions. These were based on transformations prominent in the literature for such compounds,^18,19^ enabling easy development in future, and operated on opposite ends of the molecule to provide initial independent evaluation of modification sites. Acetylating the alcohols at positions 2, 3, and 23 gave **MA-2**, lowering polarity from Clog *P* of **MA** at 3.97 to 6.64. Additionally, conversion of the carboxylic acid into an amide using 1,10-diaminodecane to add a hydrophobic chain and introduce a cationic group (likely to interact with bacterial lipids) gave **MA-3** (Clog *P* = 8.63). We also produced the 1,10-diaminodecane amide without acetylation (**MA-4**, Clog *P* = 5.96) to create a double-mutant cycle. These compounds have been previously studied for anticancer activity, and were synthesised in the same manner, starting from **MA**.^18^ As before, these were docked into the quinol binding site of cytochrome *bd*-I (Fig. 3). Compared with the predicted *K*_d_ of 1.14 μM for **MA**, acetylation resulted in a slightly stronger affinity (*K*_d_ = 0.96 μM) for **MA-2**. However, when the long chain amine was also added to give **MA-3**, the affinity was substantially weakened (*K*_d_ = 14.4 μM). The chain appears to add too much bulk, and impedes the binding geometry. Removal of some of the molecule's steric bulk in the unacetylated **MA-4** resulted in a moderate improvement in *K*_d_ (8.64 μM). Importantly, all of these are modelled to bind more strongly than **UQ-8**, and therefore we could expect inhibitory activity.

**Fig. 4:**
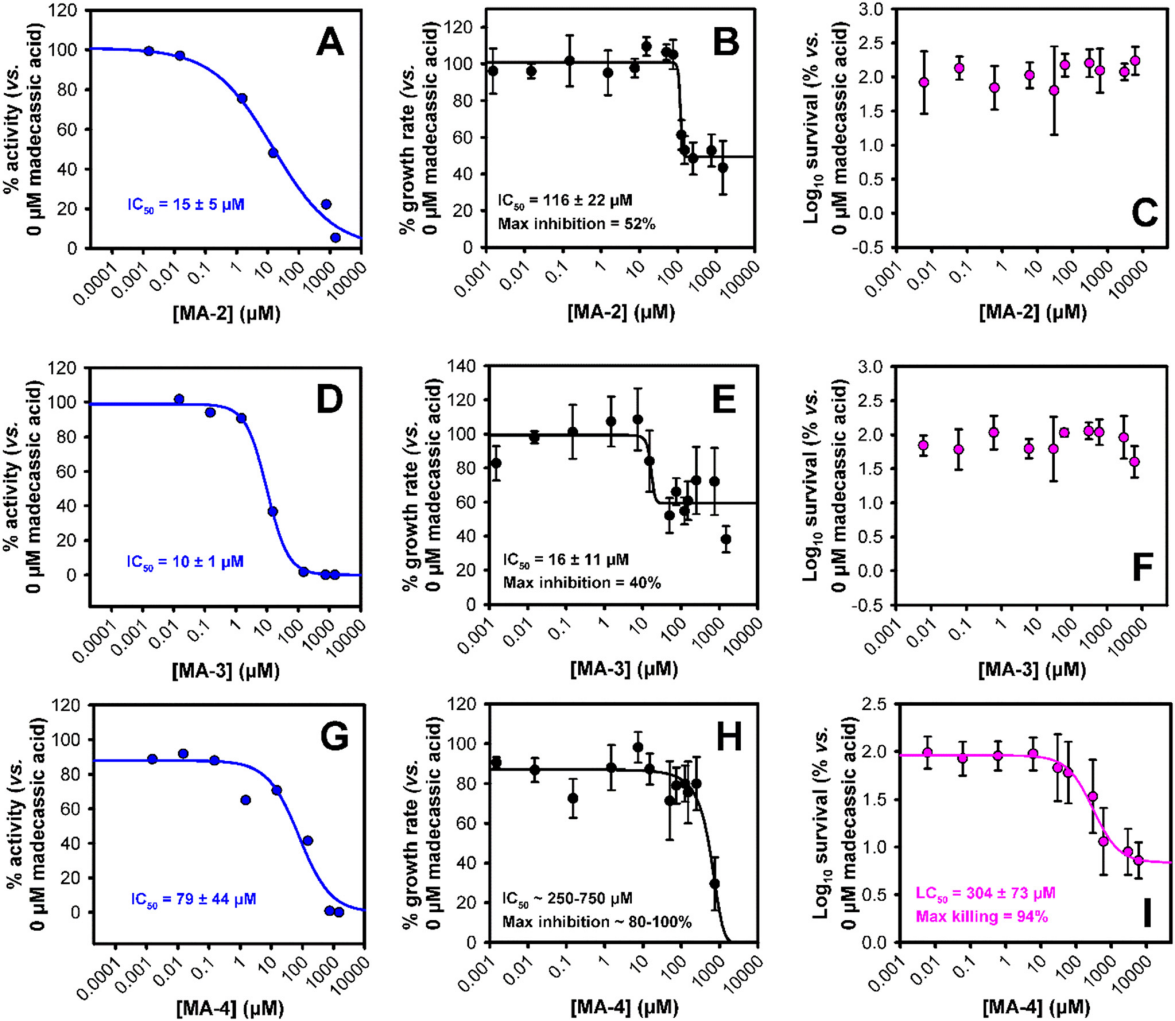
Impact of madecassic acid derivatives upon *E. coli* growth, cytochrome *bd*-I activity, and *E. coli* survival. (A, D and G) Oxygen consumption activities of *E. coli* EC958 cytochrome *bd*-I-only membranes exposed to madecassic acid derivatives. (B, E and H) Growth assays *E. coli* cytochrome *bd*-I only cells exposed to madecassic acid derivatives. (C, F and I) Survival assays for *E. coli* EC958 cytochrome *bd*-I only cells exposed to madecassic acid derivatives. Where possible, data were fitted to three- or four-parameter logistic equations using nonlinear regression (Sigmaplot) to generate IC_50_ values (or LC_50_ for killing data) with standard error. Error bars represent standard deviations for at least four technical repeats, including two biological repeats.

Oxygen consumption assays were then performed with *bd*-I-only membranes (Fig. 4a). The tighter predicted binding of **MA-2** compared with **MA** correlated with elevated inhibition (IC_50_ = 15 μM, Fig. 4A), as did the slightly weaker binding of **MA-4** correspond to a lowered inhibition (IC_50_ = 79 μM, Fig. 4G). However, **MA-3** was more active than would be expected from the predicted binding (IC_50_ = 10 μM, Fig. 4D). This is potentially due to the more lipophilic nature of that compound which may result in it being drawn into the membranes. The same compound performed well in the growth inhibition of *bd*-I only *E. coli* cells (IC_50_ = 16 μM, Fig. 4E), although was not as potent as **MA** (IC_50_ = 9.4 μM, Fig. 2B). **MA-2** was much less active (IC_50_ = 116 μM, Fig. 4B) despite having good activity against membranes, while **MA-4**′s weak performance against membranes was echoed in its limited (IC_50_ > 250 μM, Fig. 4H) growth inhibition. In light of these results, it was therefore a surprise when **MA-4** revealed itself as the only compound tested which had any capacity (albeit weak) to kill *E. coli bd*-I only cells (Fig. 4I), with an LC_50_ of 304 μM.

## Discussion

These results show that inhibitory activity of bacterial cytochromes is involved in the antibacterial activity of **MA**, with binding to the ubiquinol site of cytochrome *bd*-I being of particular interest, to which **MA** and all our derivatives are modelled to bind more strongly than the natural substrate. In line with these results, **MA** itself shows inhibition of enzymatic activity and bacterial growth, although without any observed killing of *E. coli* – this is consistent with other studies which found that Gram-positive bacteria were more vulnerable.^5^

Having applied chemical modifications to **MA**, we were able to improve enzymatic inhibition activity in membrane assays for two out of three variants (**MA-2** and **MA-3**). Unmodified **MA** proved best for growth inhibition, although **MA-3**, the most highly modified version was close behind, despite being expected to bind the protein the least strongly. However, it was only **MA-4**, ranked third in predicting binding and last in enzymatic effects, which was found to kill *E. coli*. It must be said therefore that the links between predicted binding, and the different levels of biological activity (protein activity in isolated membranes, growth inhibition, cell killing) are mixed. This supports previous work by other groups which have identified a variety of mechanisms through which **MA** has antibacterial effects including membrane disruption, inhibition of protein synthesis, and topoisomerase inhibition.^6^ In particular, we note that our modifications make the compound significantly more hydrophobic as reflected in a shift of calculated partition coefficient of four log_10_ units between **MA** and **MA-3**, as well as swapping an anionic (at biological pH) carboxylate for a cationic ammonium; both of these changes are likely to increase interactions with lipids. The proteins of interest reside in the inner membrane of *E. coli*, and their natural substrate is primarily found in the lipid bilayer. This increased lipidic interaction could therefore both increase the local concentration of **MA** derivatives, or conversely result in competitive sequestration within the lipid bilayer, away from the protein. Convolution of these effects with the other mechanisms at play have led to the overall result that **MA-4** has the greatest bactericidal activity. The myriad ways in which natural products such as **MA** can interact with biological macromolecules leads to complications which are hard to avoid – in our case, the possibility that **MA** might interact with succinate dehydrogenase^5^ was a potential limitation for the isolated membrane components of this study, since the oxygen consumption assay relies upon this complex to maintain the supply of ubiquinol for cytochrome *bd*-mediated oxygen consumption. It was therefore necessary to perform additional control experiments to exclude **MA**-mediated SDH inhibition and further demonstrate the inhibition of cytochrome *bd*-I activity where electron delivery is independent of SDH. To introduce an additional level of rigour to these investigations, an alternative *bd*-I only strain was engineered where an oxidase null mutant strain was complemented with a plasmid encoding the cytochrome *bd* operon (SI). Membranes from this *E. coli* EcoM4 pSU2718G-*cydABX*-his_6_ strain were prepared and SDH activity measurements confirmed that 1 mM MA did not inhibit SDH in isolated membranes (Fig. S2). To verify that MA directly targets cytochrome *bd*-I directly, oxygen consumption experiments were undertaken using the same membranes and duroquinol as the electron donor (delivers electrons directly to *bd*-I). These data (Fig. S3) confirmed that 1 mM MA completely abolishes *bd*-I activity, further supporting the direct binding of MA to *bd*-I in EC958 membranes.

Plants have evolved a certain set of natural products which have a particular role for that plant. Because they interact with the biological machinery in one organism, there is a good chance that they will do something in other organisms, and this has historically led to many successful medicines.^20^ However, there is no reason to expect natural products to behave exactly as we would wish, and off-target effects are common, since the compounds were selected for a different role. Chemical modification can make a big difference,^21^ and we have shown here that modifications can alter the behaviour of **MA** in bacterial models, and this provides a route to focus the activity of a natural product into a particular mechanism. Through strategic chemical modification, it should therefore be possible to both better understand the different mechanisms of triterpenoid antibacterial activity, and create molecules which maximise their potency against specific targets. Cytochrome *bd* oxidases are particularly attractive in this regard since they are only found in prokaryotic organisms. Given the versatile modification chemistry open to madecassic acid,^18,19,22,23^ further work to maximise its activity against these proteins would be well justified.

## Conclusion

Madecassic acid and three derivatives thereof have been modelled to, and shown to have, a high affinity for cytochrome *bd*-I oxidase, and experimental observations of enzymatic activity, bacterial growth, and bactericidal assays are consistent with this modelling. Alterations of the chemical structure have been shown to influence the biological activity, although the relationships between the different assay outcomes are non-linear. These findings show that inhibition of cytochrome *bd* oxidases is a further component of the antibacterial activity of madecassic acid, in addition to mechanisms already investigated by others. Further strategic chemical modification could result in new antimicrobial compounds based upon madecassic acid or other similar plant-derived compounds, which are specifically targeted towards the unique aspects of bacterial biochemistry.

## Author contributions

All authors contributed to writing the manuscript. TTPT extracted and purified the MA from *C. asiatica*. GNS and HICDS performed organic transformations. SAH was involved in planning and performing the microbiological experiments and data analysis. RAB developed the duroquinol assay, CMW engineered the complementation vector, GJ performed the SDH assays and duroquinol experiments, GKR contributed to the planning of the microbiological experiments and data analysis. CJS and MDG obtained funding and supervised the chemistry. MS also obtained funding and was responsible for all microbiological experimental design and data analysis.

## Conflicts of interest

There are no conflicts of interest.

## Supplementary Material

MD-017-D5MD01116G-s001

## Data Availability

Processed data for this paper is included in the main manuscript. Raw data for this paper (molecular characterisation, assay readout) is available from the authors upon request. Supplementary information (SI) is available. See DOI: https://doi.org/10.1039/d5md01116g.
