# Supplementary material for: Investigating the role of cytochrome bd oxidases in the antibacterial activity of madecassic acid and derivatives thereof
Source: RSC Med Chem. 2026 Jan 13;17(3):1513–20. doi: 10.1039/d5md01116g (PMC12822377; doi:10.1039/d5md01116g)
Supplement: MD-017-D5MD01116G-s001 [file MD-017-D5MD01116G-s001.pdf]

Supplementary Information (SI) for RSC Medicinal Chemistry.
This journal is © The Royal Society of Chemistry 2026

Investigating the role of cytochrome *bd* oxidases in the antibacterial activity of madecassic acid and derivatives thereof

Samantha A. Henry,[a] Geraud N. Sansom,[a] Thao Thi Phuong Tran,[b] Ryan A. Boughton,[a] Guy Joiner,[a] Calum M. Webster,[a] H. Ireshika C. de Silva,[c] Michelle D. Garrett,[a] Christopher J. Serpell,[d] Gary K. Robinson,[a] Mark Shepherd[a]

[a] School of Natural Sciences, University of Kent, Canterbury, United Kingdom, CT2 7NH, UK
[b] Institute of Chemistry, Vietnam Academy of Science and Technology, 18 Hoang Quoc Viet Road, Cau Giay, Hanoi, Vietnam
[c] Department of Chemistry, University of Colombo, Colombo 03, Sri Lanka
[d] School of Pharmacy, University College London, 29-39 Brunswick Square, London, WC1N 1AX, UK

SUPPLEMENTARY METHODS

Complementation of a respiratory null E. coli mutant with cytochrome bd-I

To independently verify that madecassic acid targets cytochrome *bd*-I of *E. coli* (and not succinate dehydrogenase that feeds the quinol pool in the activity assays), the *cydABX* operon was amplified from *E. coli* EC958 via colony PCR using the following primers and cloned into the pSU2718G vector previously used(1) by the host lab:
5'- CGGGGATCCTTAAAGAGGAGAAAGGTACATGATGTTAGATATAGTCGAACTGTCGCG-3'
5'-ATGCCTGCAGGTCGACTCTAGAAATTCAATGGTGATGGTGATGGTGGATGTCTTCTTGACCGGCTTTGCCTGATTC-3'.
The Gibson Assembly primers introduced DNA encoding a his-$_6$ tag onto the C-terminus of *cydX* gene, and successful cloning was confirmed using Sanger sequencing. The EcoM4 *E. coli* respiratory mutant strain(2), which lacks all respiratory oxidases, was transformed with the pSU2718G-*cydABX*-his$_6$ vector and expression of cytochrome *bd*-I was confirmed using CO difference spectroscopy in whole cells to detect a *d*-type haem signal, as previously described(3).

Succinate dehydrogenase assays

The method used for the succinate dehydrogenase assay is an adaptation of Kolaj-Robin et al. 2011(4). Assays (700 µL) comprised 50 mM Tris-HCl (pH 8.5), 400 µM phenazine methosulfate (PMS), 8 mM succinate, 0.02 % n-dodecyl-β-D-maltoside, 50 µg/mL of *E. coli* EC958 membranes and 50 µM of dichlorophenolindophenol (added last). Assays were set up in the presence and absence of 1 mM madecassic acid (dissolved in DMSO). Assay mixtures were preincubated for 3 min at 37 °C before being started via addition of the DCPIP followed by rapid mixing with a pipette. Spectrakinetics were recorded on a Cary 60 spectrophotometer for 15 min and rates were calculated using the extinction coefficient A_{600} = 20.7 $mM^{-1}cm^{-1}$ (5).

Oxygen electrode cytochrome bd-I assays with duroquinol as the electron source

Membranes from the *E. coli* EcoM4 pSU2718G-*cydABX*-his_6 strain were prepared as described previously(6). To specifically monitor cytochrome *bd*-I activity, duroquinol was used as a directly electron source. Duroquinol was prepared from duroquinone as previously described(7) and quantified using the extinction coefficient $\Delta A_{275(ox-red)} = 16\ mM^{-1}cm^{-1}$ (8). Cytochrome *bd*-I activity was then monitored via oxygen consumption in a Rank Brothers oxygen electrode with a 2 mL closed chamber at 37 ^{o}C, which contained 50 mM HEPES pH 7.4, 0.5 mg/mL membranes (based on wet membranes) and DMSO-solubilised drug (added from 40x final concentration) or 'DMSO only' control. A final concentration of 400 µM duroquinol (in 50 mM KPi, 2mM EDTA pH 7.5) was added to initiate the reaction.

REFERENCES

1. Shepherd M, Achard MES, Idris A, Totsika M, Phan MD, Peters KM, et al. The cytochrome bd-I terminal oxidase of uropathogenic *Escherichia coli* augments survival during infection. *Sci Rep*. **2016**; 6 (35285). doi:10.1038/srep35285
2. Portnoy VA, Scott DA, Lewis NE, Tarasova Y, Osterman AL, Palsson BØ. Deletion of genes encoding cytochrome oxidases and quinol monooxygenase blocks the aerobic-anaerobic shift in *Escherichia coli* K-12 MG1655. *Appl Environ Microbiol*. **2010**;76(19):6529–40. doi:10.1128/AEM.01178-10
3. Holyoake LV, Hunt S, Sanguinetti G, Cook GM, Howard MJ, Rowe ML, et al. CydDC-mediated reductant export in Escherichia coli controls the transcriptional wiring of energy metabolism and combats nitrosative stress. *Biochem J*. **2016**;473(6):693–701. doi:10.1042/BJ20150536
4. Kolaj-Robin O, O'Kane SR, Nitschke W, Léger C, Baymann F, Soulimane T. Biochemical and biophysical characterization of succinate:quinone reductase from *Thermus thermophilus*. *Biochim Biophys Acta Bioenerg.* **2011**;1807(1):68–79. doi:10.1016/j.bbabio.2010.10.009
5. Hägerhäll C, Aasa R, von Wachenfeldt C, Hederstedt L. Two hemes in Bacillus subtilis succinate:menaquinone oxidoreductase (complex II). Biochemistry. **1992**;31(32):7411–21. doi:10.1021/bi00147a028
6. Henry SA, Webster CM, Shaw LN, Torres JT, Jobson ME, Totzke BC, et al. Steroid drugs inhibit bacterial respiratory oxidases and are lethal towards methicillin-resistant *Staphylococcus aureus*. *J Infect Dis.* **2024**;230(1):e149–58. doi:10.1093/infdis/jiad540
7. Rich P. Electron transfer reactions between quinols and quinones in aqueous and aprotic media. *Biochim Biophys Acta.* **1981**;637(1):28–33. doi:10.1016/0005-2728(81)90206-1
8. Degli Esposti M, Lenaz G, Izzo G, Papa S. Kinetics and sidedness of ubiquinol-cytochrome c reductase in beef-heart mitochondria. *FEBS Lett.* **1982**;146(1):101–5. doi:10.1016/0014-5793(82)80713-8

SUPPLEMENTARY DATA

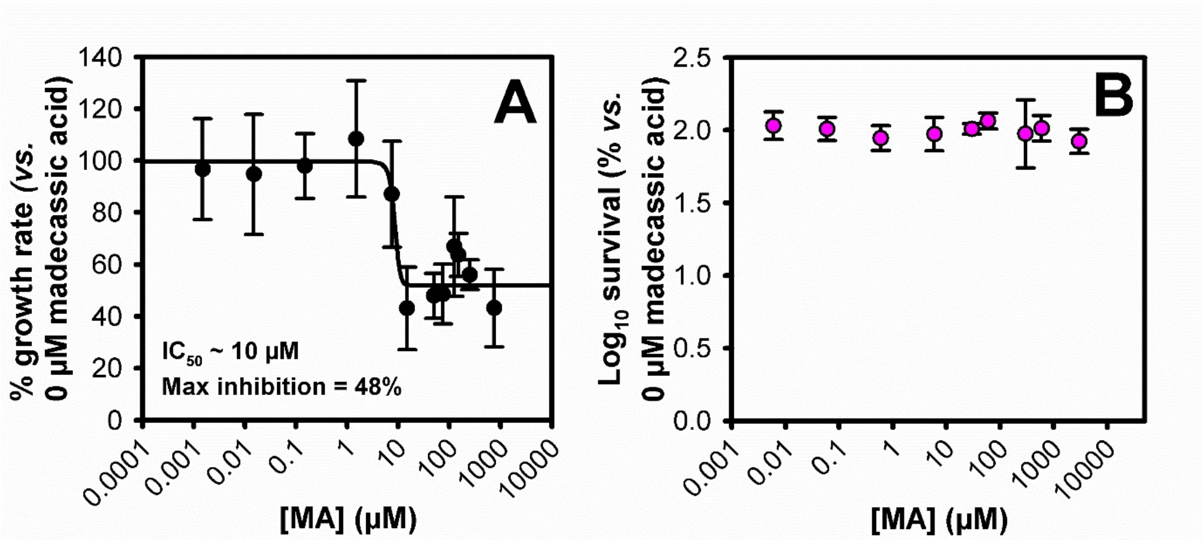

Figure S1. Impact of madecassic acid upon *E. coli* growth and survival. (A) Growth assays of MA against wild type *E. coli* EC958 cells. Error bars represent standard deviations for at least 4 technical repeats, including two biological repeats. (B) Wild type *E. coli* EC958 cells were exposed to various concentrations of madecassic acid and bacterial survival was enumerated using serial dilution and viability plating. Error bars represent standard deviations for at least four technical repeats, including two biological repeats.

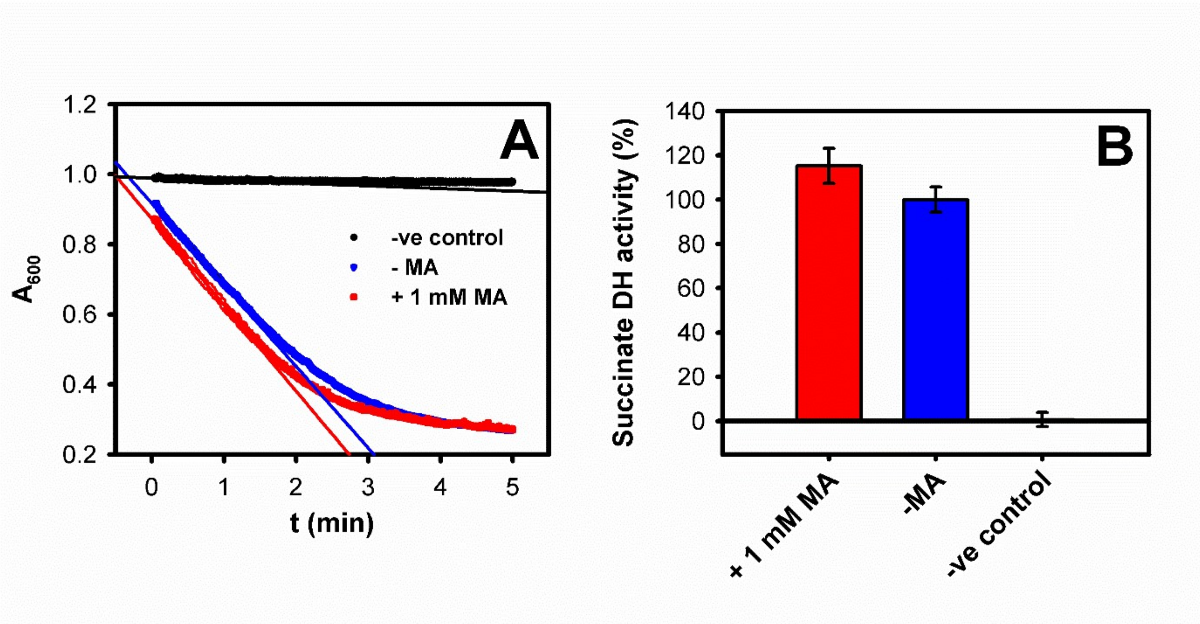

Figure S2. Madecassic acid does not diminish succinate dehydrogenase activity. Isolated *E. coli* EC958 membranes were assayed for succinate dehydrogenase activity in the presence and absence of 1 mM madecassic acid (MA), and negative controls were recorded in the absence of membranes. (A) representative raw data traces for positive control (-MA), negative control (-ve control and test conditions with madecassic acid (+1 mM MA). (B) Rates were averaged from a minimum of three repeats and error bars show the standard deviation. Data are normalised to display the positive control data as 100%.

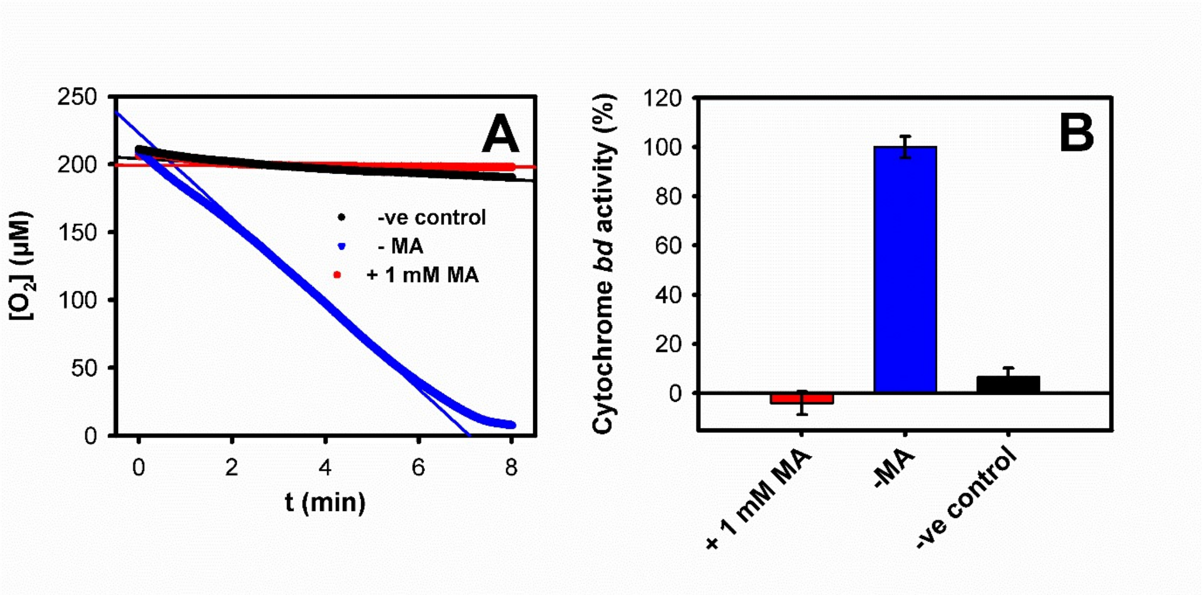

Figure S3. Madecassic acid abolishes cytochrome *bd* activity in a respiratory null *E. coli* mutant complemented with cytochrome *bd*-I. Isolated *E. coli* EcoM4 pSU2718G-*cydABX*-his$_6$ membranes (respiratory null mutant with *cydABX* on a complementation vector) were assayed for cytochrome *bd*-I activity with duroquinol supplying the electrons. Cytochrome *bd*-I activity was measured via monitoring oxygen consumption in an oxygen electrode in the presence and absence of 1 mM madecassic acid, and negative controls were recorded in the absence of membranes. (A) representative raw data traces for positive control (-MA), negative control (-ve control and test conditions nwith madecassic acid (+1 mM MA). (B) Rates were averaged from a minimum of two repeats and error bars show the standard deviation. Data are normalised to display the positive control data as 100%.
